# A new FRET-based platform to track substrate ubiquitination by fluorescence

**DOI:** 10.1074/jbc.RA120.016858

**Published:** 2021-01-07

**Authors:** Kenneth Wu, Kevin Ching, Robert A. Chong, Zhen-Qiang Pan

**Affiliations:** Department of Oncological Sciences, The Icahn School of Medicine at Mount Sinai, New York, New York, USA

**Keywords:** protein degradation, ubiquitination, E3 ubiquitin ligase, FRET, kinetics, chemoenzymatic ligation, βTrCP, beta-transducin repeats-containing protein, Aux, auxin, Cdc34, cell division cycle 34, CRBN, cereblon, CRL4, Cullin-RING E3 ubiquitin ligase 4, CUL1, Cullin1, E1, E1 ubiquitin-activating enzyme, E2, E2 ubiquitin-conjugating enzyme, E3, E3 ubiquitin ligase, Fbxw7, F-box/WD repeat-containing protein 7, GST, glutathione-*S*-transferase, HTS, high-throughput screening, IκBα, nuclear factor of kappa light polypeptide gene enhancer in B-cells inhibitor, alpha, Myc, myelocytomatosis, ROC1, RING-box protein 1, SCF, Skp1–Cullin1–Fbox protein E3 ligase, Skp2, S phase kinase-associated protein 2, TCEP, tris(2-carboxyethyl)phosphine, TIR1, transport inhibitor response 1, UB, ubiquitin

## Abstract

Post-translational modification of protein by ubiquitin (Ub) alters the stability, subcellular location, or function of the target protein, thereby impacting numerous biological processes and directly contributing to myriad cellular defects or disease states, such as cancer. Tracking substrate ubiquitination by fluorescence provides opportunities for advanced reaction dynamics studies and for translational research including drug discovery. However, fluorescence-based techniques in ubiquitination studies remain underexplored at least partly because of challenges associated with Ub chain complexity and requirement for additional substrate modification. Here we describe a general strategy, FRET diubiquitination, to track substrate ubiquitination by fluorescence. This platform produces a uniform di-Ub product depending on specific interactions between a substrate and its cognate E3 Ub ligase. The diubiquitination creates proximity between the Ub-linked donor and acceptor fluorophores, respectively, enabling energy transfer to yield a distinct fluorescent signal. FRET diubiquitination relies on Ub–substrate fusion, which can be implemented using either one of the two validated strategies. Method 1 is the use of recombinant substrate–Ub fusion, applicable to all substrate peptides that can bind to E3. Method 2 is a chemoenzymatic ligation approach that employs synthetic chemistry to fuse Ub with a substrate peptide containing desired modification. Taken together, our new FRET-based diubiquitination system provides a timely technology of potential to advance both basic research and translation sciences.

Post-translational modification of protein by ubiquitin (Ub) alters the abundance, subcellular location, or function of the target protein in a profound fashion that impacts numerous biological processes and directly contributes to myriad cellular defects or disease states, such as cancer (1, 2). The biochemical process of covalently attaching Ub to a protein substrate, termed ubiquitination, is driven by three enzymes: the E1-activating enzyme, E2-conjugating enzyme, and E3 ligase that recognize a substrate and hence, governs specificity of the reaction (1, 3). The results of biochemical analysis and atomic resolution structural studies have shown that a complex of E3 and substrate nucleates a multicomponent assembly containing substrate, E3, E2, and Ub (4). The resulting multifaceted protein–protein interactions promote the alignment of the substrate's receptor lysine residue to the donor Ub's G76, thereby enabling a nucleophilic attack that yields an isopeptide bond linkage. In a subsequent step that leads to polyubiquitination, the substrate-linked Ub serves as a receptor that provides a lysine residue to attack the incoming E2∼donor Ub thiol ester complex, forming an Ub–Ub chain.

Many oncoproteins or tumor suppressors are substrates of the Ub–proteasome system (2, 5, 6). Evidence emerges to suggest that therapeutic perturbation of ubiquitination may be desirable for cancer therapeutics. To this end, tracking substrate ubiquitination by fluorescence provides opportunities for translational research including drug discovery. For instance, fluorescence-based tracking of ubiquitination can be utilized in high-throughput screening (HTS) that may result in identification of small-molecule modulators. Conceptually, on the one hand, fluorescent ubiquitination-based functional HTS can be used to search for a small-molecule antagonist that would block ubiquitination of a tumor suppressor, thereby elevating the tumor suppressor protein level to enhance tumor inhibition activity. This is exemplified by the ability of Nutlin to block the activity of E3 Mdm2 to target p53 (7). Conversely, a small-molecule agonist could accelerate the degradation of an oncogene product. Lenalidomide acts to increase the ability of E3 Cullin-RING E3 ubiquitin ligase 4 (CRL4^CRBN^) to target Ikaros family zinc finger protein (8, 9) and casein kinase 1α (10).

Despite repeated efforts (11, 12, 13, 14, 15, 16, 17, 18), however, fluorescence-based ubiquitination assays are an underdeveloped area at least partly because of several challenges associated with the complexity of ubiquitination. First, substrate–E3 interaction is often triggered by post-translational modification of the target protein, such as phosphorylation (5, 6). Enzymatic modification reaction, such as phosphorylation of nuclear factor of kappa light polypeptide gene enhancer in B-cells inhibitor, alpha (IκBα) by IKKβ (19), can be of exceedingly high cost and therefore unfeasible for a biochemical HTS campaign. It is thus of timely importance to develop a general strategy that enables generation of a substrate in a state competent for binding to its cognate E3 without the need of enzymatic modification. The second challenge is the placement of fluorophore. Previous studies have developed FRET-based *in vitro* reporter assays using N-terminally labeled Ub (11, 15, 16, 17). However, such fluorescent Ub may not be optimal for FRET detection of linkage-specific Ub chain formation (20). In addition, detection of fluorescent signal is often *via* indirect approaches, typically through antibody-aided methods (12, 13, 14). The indirect FRET techniques, however, may result in undesirable signal/background ratio. Finally, ubiquitination typically produces chains of heterogeneous size, and this complexity may compromise reproducibility. Thus, it is desirable to devise a strategy that yields a uniform Ub product to ensure a high degree of reproducibility critical to HTS success.

To address these challenges, we developed a novel “FRET diubiquitination” system that utilizes recombinant fusion or chemoenzymatic ligation strategy to generate a fusion between Ub and substrate. We demonstrate that such a fusion substrate is active, allowing measurement of E3-dependent diubiquitination by fluorescence in real time.

## Results

### Design FRET diubiquitination

**Figure 1:**
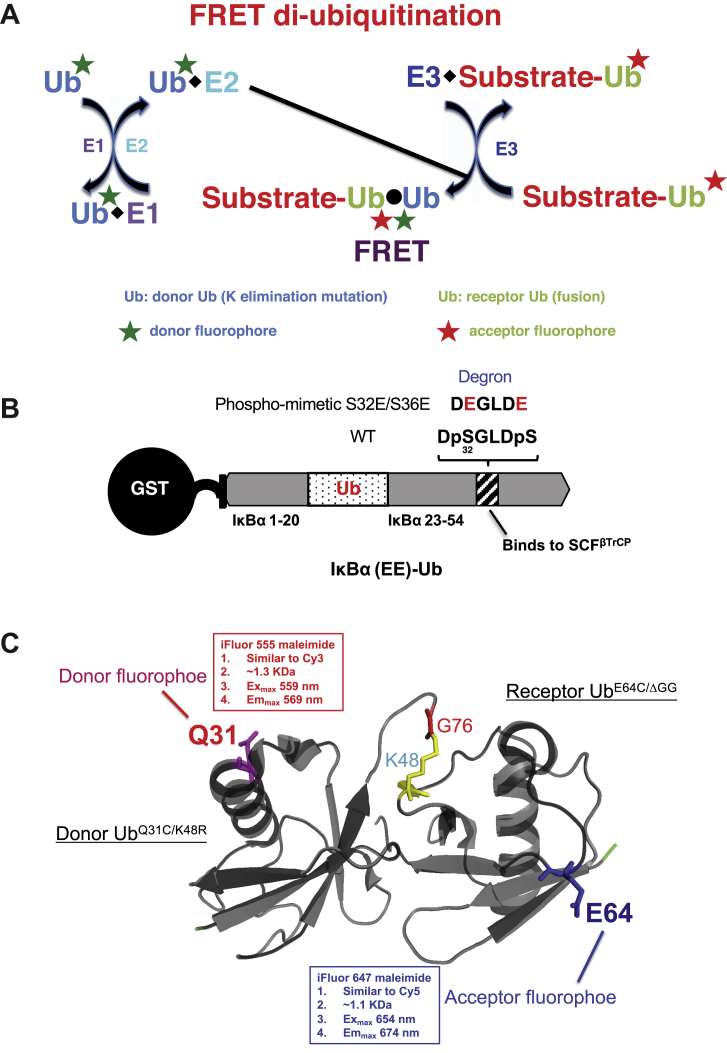
**FRET diubiquitination design.***A*, general scheme. Diubiquitination is a result of mixing two reaction assemblies. On the *right* hand, a substrate–receptor Ub fusion, in which Ub is labeled with *red*-coded acceptor fluorophore, binds to its cognate E3 to form the E3/substrate–Ub complex (assembly 1). On the *left* hand, a donor Ub, containing a lysine substitution that eliminates Ub chain assembly, is labeled with *green*-coded donor fluorophore. This donor Ub reacts with E1 and E2 to form E2∼donor Ub thiol ester complex (assembly 2). The combination of assemblies 1 and 2 produces a uniform substrate–di-Ub product, resulting in close proximity between the *red* and *green* fluorophores, respectively. When the donor fluorophore is excited, its emission causes the excitation of the acceptor fluorophore, which emits a distinct fluorescent signal. *B*, Ub fusion with IκBα phosphomimetic degron. IκBα (1–54) is a well-characterized N-terminal fragment of IκBα that contains all the molecular elements required for degradation (19). It possesses a phosphodegron (DpSGLDpS) that binds to E3 SCF^βTrCP^. To mimic phosphorylation, S32 and S36 are replaced by glutamic acid (E). In addition, IκBα K21 and K22, the authentic receptors for ubiquitination, are replaced by Ub (1–74), which now provides K48 as the attacking lysine residue. This fusion is named IκBα (EE)–Ub. The N-terminal GST tag can be removed by thrombin cleavage. *C*, fluorophore placement on Ub. Donor Ub Q31 and receptor Ub E64 are substituted by cysteine and labeled with the indicated fluorescent dye. Ub, ubiquitin.

To advance ubiquitination studies using fluorescent probes, we developed FRET diubiquitination system characterized by two main features (Fig. 1*A*). First, diubiquitination is based on an Ub–substrate fusion strategy. This fusion creates a dual-function molecule. On the one hand, it contains a substrate peptide capable of interacting with its cognate E3 ligase. Owing to the ability of an E3 to recruit an E2∼donor Ub thiol ester (3), the fusion molecule can nucleate a complex of substrate–E3–E2–donor Ub. On the other hand, the substrate-linked Ub provides a receptor lysine residue to attack the E2∼donor Ub thiol ester. In addition, the donor Ub is altered by site-directed mutagenesis to eliminate an internal lysine residue required for Ub chain assembly by the select E2. As a consequence, the Ub–substrate fusion molecule directs diubiquitination only, producing a uniform di-Ub product. The second feature of the FRET diubiquitination is the placement of a pair of distinct fluorophores into the donor and receptor Ub, respectively. The juxtaposition of two Ub-linked fluorophores, as a result of diubiquitination, results in energy transfer to yield a tractable fluorescent signal.

In this work, we used a well-characterized model ubiquitination system comprising substrate IκBα, Skp1–Cullin1–Fbox protein E3 ligase (E3 SCF^βTrCP^), and E2 cell division cycle 34 (Cdc34) (19). Figure 1*B* shows design of the Ub–IκBα fusion. Human IκBα N-terminal fragment (amino acids 1–54) was used, and it contains a signature 6-amino acid phosphodegron that is known to engage affinity interactions with E3 SCF^βTrCP^ (21). To engineer a constitutively active IκBα degron, serine residues 32 and 36 were replaced by glutamic acid to mimic phosphorylation. In addition, IκBα K21 and K22, the authentic receptors for ubiquitination, were removed and replaced by Ub residues 1 to 74. The fusion product, IκBα (1–54)^S32ES36E^–Ub, is designated as IκBα (EE)–Ub. Previous studies have validated the function of IκBα (EE)–Ub, which allows ubiquitination by SCF^βTrCP^ without the need of phosphorylation by IKKβ (20). In addition, multiple lines of evidence demonstrated the function of the fusion Ub as a receptor for ubiquitination (22).

For FRET, we placed a pair of distinct fluorophores into specific sites located within the donor and receptor Ub, respectively, as illustrated in Figure 1*C*. The donor Ub carries a cysteine substitution at position Q31, and the resulting Ub C31 is linked to fluorophore iFluor 555 through maleimide-mediated coupling. Likewise, the receptor Ub is modified to contain a change at position 64 (E → C), where fluorophore iFluor 647 is placed. As shown by our recent study (20), incubation of this pair of fluorescently labeled donor and receptor Ub molecules with E1, E2 Cdc34, and an E3 subcomplex (RING-box protein 1 [ROC1]–Cullin1 [CUL1] CTD) formed a di-Ub product that generated a distinct fluorescent signal. These findings have validated the design of the fluorophore placement.

### FRET IκBα diubiquitination tracks ubiquitination in real time

FRET IκBα diubiquitination is a multistep reaction. IκBα (EE)–Ub (carrying fluorescent label iFluor 647) interacts with E3 SCF^βTrCP^ to form the E3/substrate–Ub (I647) complex. The donor Ub (K48R, carrying fluorescent label iFluor 555), designated as Ub (K48R/I555), is activated by E1 to form E1∼Ub (K48R/I555), which then reacts with E2 Cdc34 to yield Cdc34∼Ub (K48R/I555). Upon mixing SCF^βTrCP^/IκBα (EE)–Ub (I647) and Cdc34∼Ub (K48R/I555), lysine 48 (K48) from IκBα (EE)-linked receptor Ub (I647) attacks Cdc34∼Ub (K48R/I555) to form the IκBα di-Ub product. As a consequence, Ub-linked fluorophores iFluor 555 and iFluor 647 are in close proximity optimal for energy transfer (FRET). E2 Cdc34 catalyzes K48 ubiquitination specifically (22). The use of donor Ub K48R allows only *one* nucleophilic attack, producing a *single* Ub–Ub isopeptide bond.

**Figure 2:**
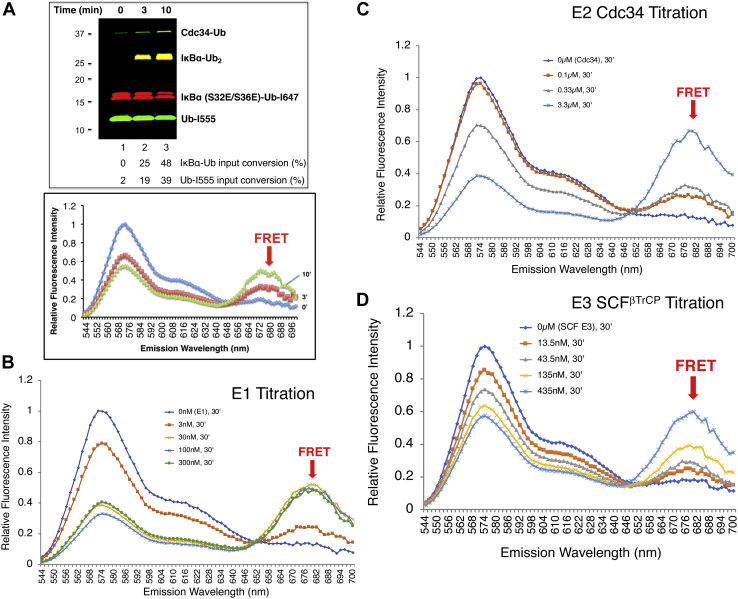
**Detection of FRET IκBα diubiquitination.***A*, FRET IκBα diubiquitination in test tube was carried out as described in Experimental procedures section. After incubation for times as indicated, aliquots of the reaction were subjected to analysis by gel electrophoresis (*top*) or spectroscopy (*bottom*). *B*–*D*, titration of E1 (*B*), E2 Cdc34 (*C*), and E3 SCF^βTrCP^ (*D*).

The reaction was first analyzed by standard gel electrophoresis followed by imaging fluorescent products with a scanner. As shown, the product IκBα–Ub_2_ increased with time (Fig. 2*A*, *top*), and >30% of the input donor Ub was converted into the product after 10 min of incubation. For FRET, a reaction aliquot was spotted onto a 384-well plate for spectroscopic analysis. FRET signal was detected, and its intensity increased with time in a manner coincidental with the abundance of IκBα–Ub_2_ detected by gel analysis (Fig. 2*A*, *bottom*). These results conclude that the observed FRET reflects the formation of IκBα–Ub_2_ as a result of enzymatic synthesis. The results of titration experiments showed that the FRET IκBα diubiquitination required E1 (Fig. 2*B*), E2 Cdc34 (Fig. 2*C*), and E3 SCF^βTrCP^ (Fig. 2*D*).

**Figure 3:**
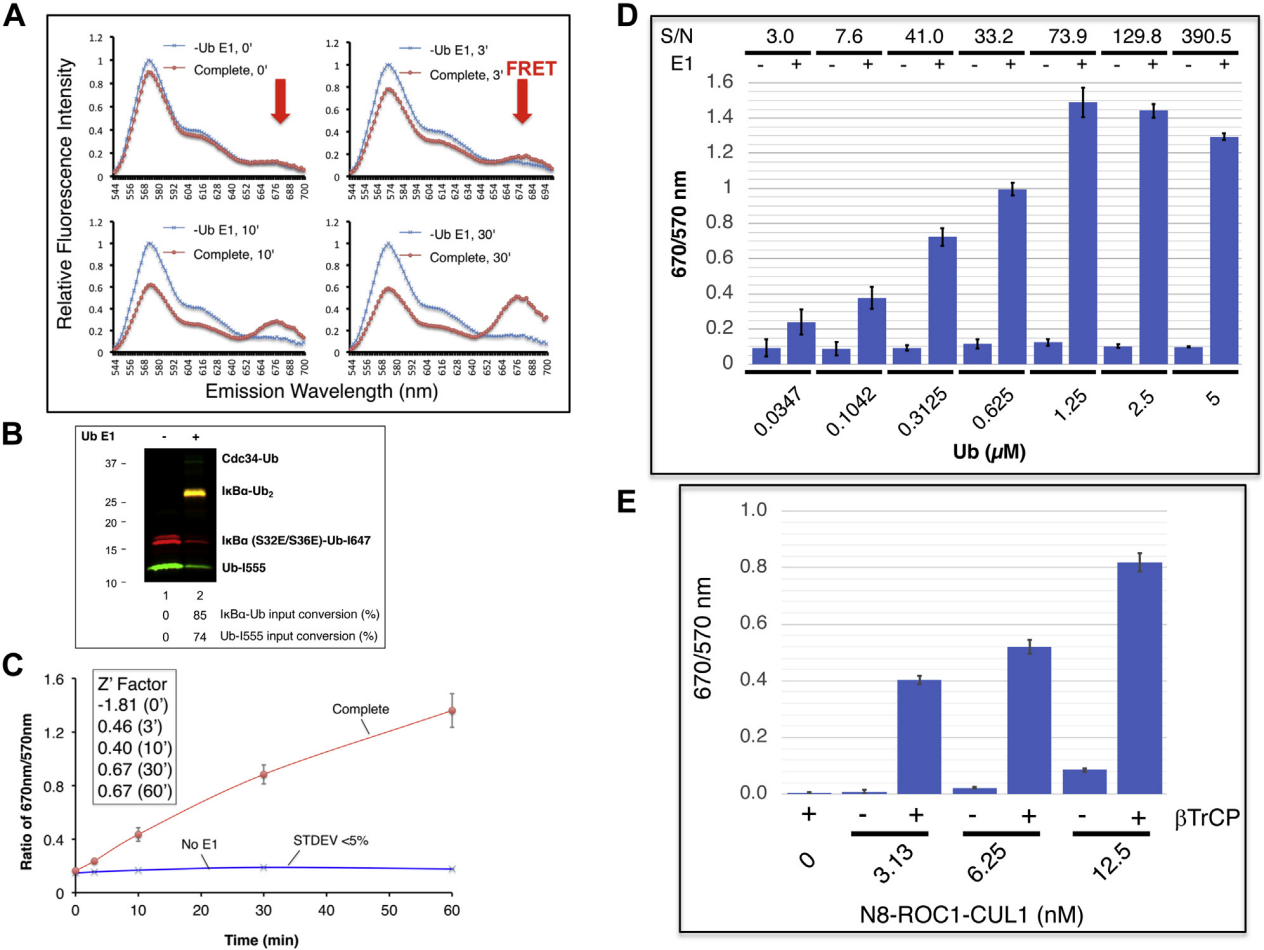
**Tracking FRET IκBα diubiquitination in real time.***A*, real-time kinetics. The real-time kinetic experiments were carried out as described in Experimental procedures section. The emission spectrum of 544 to 700 nm for the complete reaction and no E1 control are shown. *B*, gel analysis. The final reaction mixture was subject to SDS-PAGE and Typhoon FLA9500 imaging analysis. *C*, assessing reliability. Three independent real-time kinetics experiments were carried out, and the results are plotted to reveal standard deviation and calculate Z' factor. The ratio of 670/570 nm represents the FRET efficiency. Note that iFluo 647 (receptor fluorophore) emits at 670 nm, whereas iFluo 555 (donor fluorophore) emits at 570 nm. *D*, analyte titration. The analyte concentrations are as indicated. The concentrations of enzymes are as follows: E1 (50 nM), E2 Cdc34b (3 μM), and E3 Nedd8–SCF^βTrCP^ (100 nM). S/N = (mean of signal – mean of background)/standard deviation of background. The reaction at 30 °C was monitored on the fluorescence plate reader for 30 min. The 15 min time point (in linear range of the reaction) is used for graph. *E*, dependency on Skp1–βTrCP and Nedd8–ROC1–CUL1. Skp1–βTrCP (100 nM), donor/receptor Ub (2.5 μM), E2 (50 nM), Cdc34b (1 μM), IκBα (EE)–Ub (E64C)–I647 (2.5 μM), and Ub (K48R/Q31C)–I555 (2.5 μM) were used. The concentrations of Nedd8–ROC1–UL1 are as indicated. The 30 min time point (in linear range of the reaction) is used for graph. Ub, ubiquitin.

To track the FRET IκBα diubiquitination in real time, SCF^βTrCP^/IκBα (EE)–Ub (I647) and Cdc34∼Ub (K48R/I555), preassembled in separate tubes, were mixed in a 384-well plate followed by incubation at 30 °C on a fluorescence reader. Real-time fluorescence was recoded as the ubiquitination proceeded. The results revealed that FRET increased over 30 min (Fig. 3*A*). As shown by gel analysis, 85% of the input substrate–Ub was converted into the product after the reaction (Fig. 3*B*). Z' factor measures statistical effect size based on the means and standard deviations of both the positive and negative control samples (23). Three independent experiments were performed with both the complete experiment and the no E1 negative control, resulting in the determination of the Z' factor >0.5, an indication of acceptable reproducibility (Fig. 3*C*). In addition, analyte titration experiments were carried out to assess the assay's sensitivity, that is, the lower limit of detection, which is commonly defined as the threshold analyte concentration when signal-to-noise ratio equals to 3. Based on the results of the titration experiments (Fig. 3*D*), the lower limit of detection of the assay was found to be 35 nM. Finally, E3 SCF^βTrCP^ is made of two functional subcomplexes: Skp1–βTrCP that targets substrates such as IκBα, and Nedd8–ROC1–CUL1 that functions to recruit E2 Cdc34∼Ub for catalysis (19). To determine the dependency of the reaction on substrate targeting by E3, we performed an Nedd8–ROC1–CUL1 titration experiment in the presence or the absence of Skp1–βTrCP. The results revealed requirements for both subcomplexes (Fig. 3*E*). Combining with the results of the E1/E2 Cdc34 titration experiments (Fig. 2, *B*–*C*), these findings established the specificity of the FRET IκBα diubiquitination assay.

### Characterization of the FRET IκBα diubiquitination

**Figure 4:**
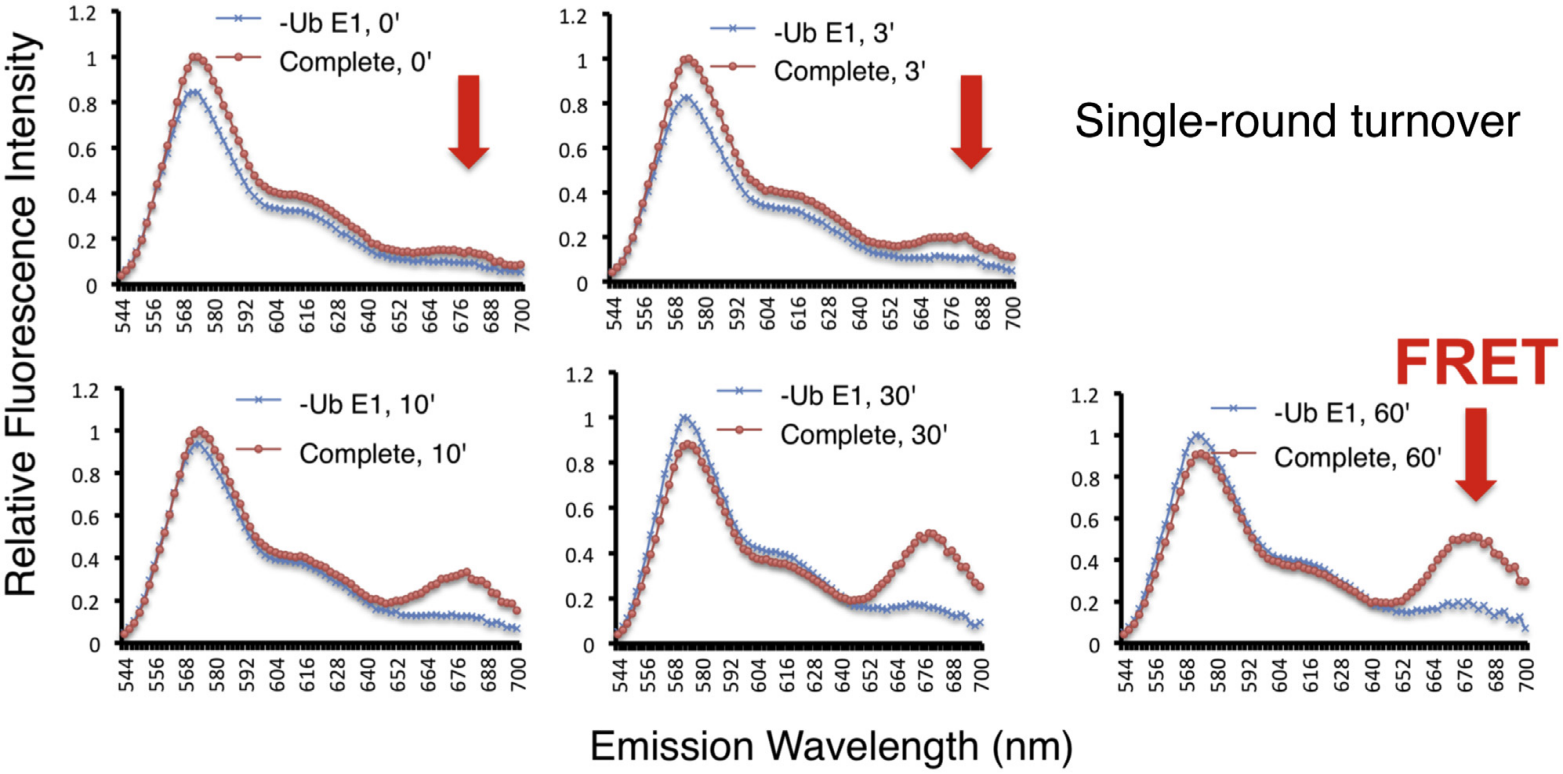
**Real-time kinetics of FRET IκBα diubiquitination under single-round turnover condition.** The reaction was carried out as in Figure 3, *A*–*C* expect that the preformed substrate–E3 and donor Ub–E2 complexes were treated with apyrase (6 mU/1 μl) for 1 min at 37 °C to deplete ATP. The apyrase-treated reaction mixture was then loaded onto a 384-well plate for incubation on the Synergy-H1 reader set at 30 °C. The reaction was monitored at times as indicated. The emission spectrum of 544 to 700 nm for the complete reaction and no E1 control are shown. Ub, ubiquitin.

We assessed whether the FRET IκBα diubiquitination maintains properties characteristic of ubiquitination. To this end, we performed the FRET IκBα diubiquitination under single-round turnover condition. For this purpose, SCF^βTrCP^/IκBα (EE)–Ub (I647) and Cdc34∼Ub (K48R/I555), preassembled in separate tubes, were each treated with apyrase to deplete ATP prior to mixing for ubiquitination. The effectiveness of ATP depletion by the apyrase protocol was validated previously (24). The results revealed that FRET plateaued at 30 min, utilizing about 20% of substrate input (Fig. 4), indicating that FRET IκBα diubiquitination indeed proceeds under the single-round turnover conditions.

**Figure 5:**
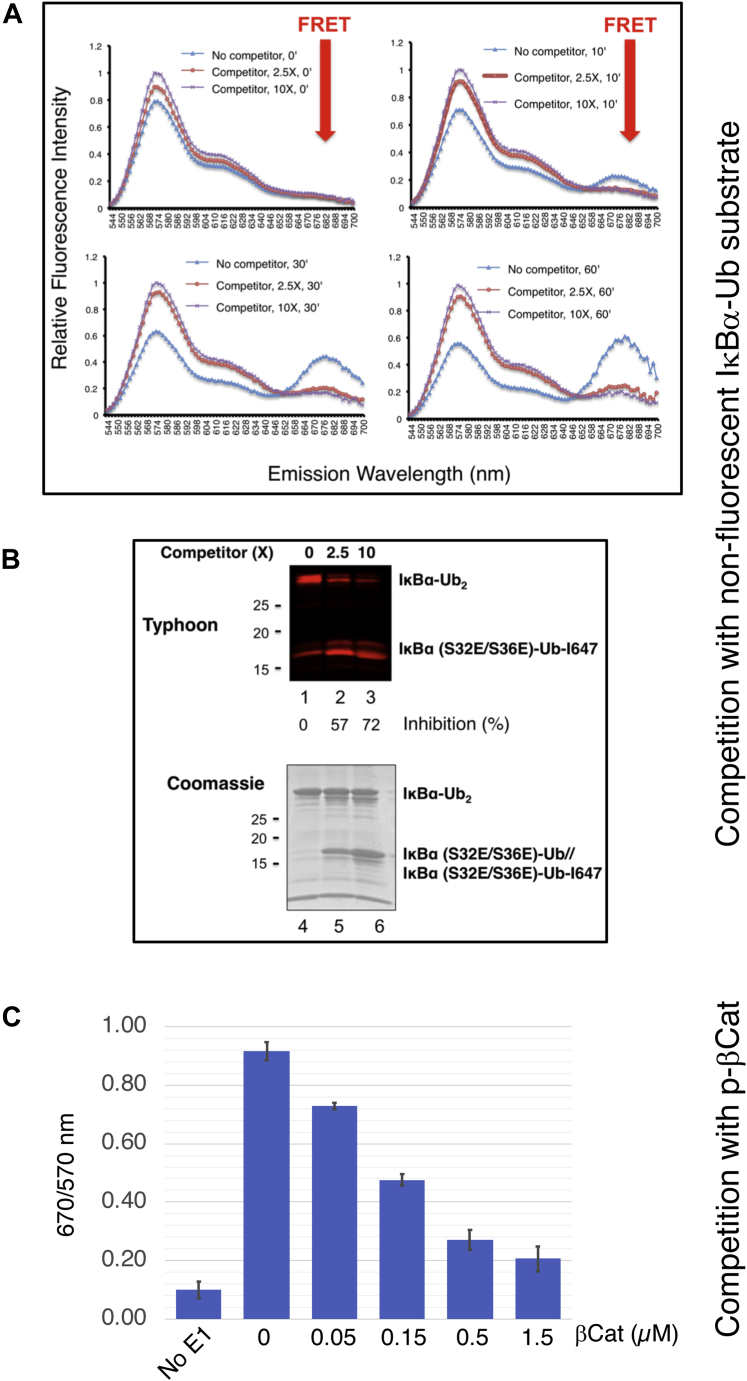
**Real-time kinetics of FRET IκBα diubiquitination with competitor.***A*–*B*, the reaction was carried as in Figure 3, *A*–*C*, except that nonfluorescent IκBα (EE)–Ub (E64C) was added 2.5× or 10× more than fluorescent IκBα (EE)–Ub (E64C)–I647. The reaction was analyzed by spectroscopic analysis (panel *A*), and gel electrophoresis followed by imaging (*top*) or Coomassie stain (*bottom*). The results showed that fluorescent IκBα–Ub_2_ product was decreased in the presence of the competitor (*top*, lanes 1–3). Coomassie staining revealed that nonfluorescent IκBα (EE)–Ub (E64C) was able to support ubiquitination, forming nonfluorescent IκBα–Ub_2_ (*bottom*, lanes 4–6). *C*, competition with β-catenin. The concentrations of enzymes and proteins are as follows: donor/receptor Ub (0.5 μM), E1 (50 nM), E2 Cdc34b (1 μM), Skp1–βTrCP (100 nM), Nedd8–ROC1–CUL1 (12 nM), IκBα (EE)–Ub (E64C)–I647 (0.5 μM), and Ub (K48R/Q31C)–I555 (0.5 μM). The reaction at 30 °C was monitored on the fluorescence plate reader for 30 min. The 18 min time point (in linear range of the reaction) is used for graph. Ub, ubiquitin.

We then assessed competitive inhibition of the FRET IκBα diubiquitination. Competition experiments were performed using nonfluorescent substrate IκBα (EE)–Ub (E64C) as competitive inhibitor. As shown, the nonfluorescent substrate, in amount 2.5 times in excess to the labeled substrate, almost completely blocked FRET (Fig. 5*A*). Gel analysis showed that excess nonfluorescent substrate inhibited consumption of the labeled input substrate (Fig. 5*B*, lanes 1–3). On the other hand, the nonfluorescent substrate was active, yielding IκBα–Ub_2_ as visualized by Coomassie stain (Fig. 5*B*, lanes 4–6). To confirm this effect, we have performed competition experiments with the phospho-β catenin degron peptide as a competitor. Previous crystallographic and biochemical studies have shown binding of the phospho-β catenin degron peptide to βTrCP (21) and ability of this peptide to support ubiquitination by SCF^βTrCP^ (24). As shown, the phospho-β catenin degron peptide was able to effectively compete against the fluorescent IκBα substrate by blocking the FRET IκBα diubiquitination (Fig. 5*C*). In all, these results suggest that the use of fluorescence did not alter reaction properties.

### Chemoenzymatic ligation and FRET β-catenin diubiquitination

**Figure 6:**
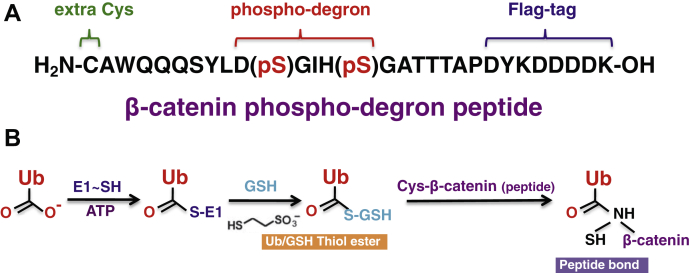
**Ligation of the β-catenin degron peptide to Ub.***A*, the β-catenin degron peptide. The β-catenin degron peptide is shown. Phosphodegron motif, N-terminal Cys residue, and Flag-tag are indicated. *B*, ligation scheme. E1 and GSH were used to promote the ligation of the β-catenin degron peptide to Ub. Ub, ubiquitin.

The FRET IκBα diubiquitination requires the recombinant Ub fusion technique, which can apply to substrates that in the absence of modification can interact with E3, or that carry a phosphodegron but can be mimicked by amino acid substitutions, such as IκBα (EE) (Fig. 1*B*). However, the recombinant Ub fusion approach may not be applicable to many substrate degrons containing post-translational modifications that cannot be readily mimicked by amino acid substitutions. To address this issue and broaden the applicability of the FRET diubiquitination approach, we have developed an alternative strategy to generate Ub–substrate fusion (Fig. 6). In this approach, the β-catenin phosphodegron peptide (NH2-CAWQQQSYLD(pS)GIH(pS)GATTTAP-OH) (Fig. 6*A*) was linked to Ub by chemoenzymatic ligation *in vitro* (Fig. 6*B*). The chemoenzymatic method is based on modification of a previously reported strategy that couples Cys–enhanced GFP to Ub by using both E1 enzyme catalysis and chemistry (25). We chose the β-catenin phosphodegron peptide (Fig. 6*A*) because it exhibits binding to βTrCP in a previously reported cocrystallographic study (21), supports efficient *in vitro* ubiquitination by SCF^βTrCP^ (24, 26), and acts as an effective competitor for the FRET IκBα diubiquitination (Fig. 5*C*). Note that the D(pS)GIH(pS) motif within this peptide (Fig. 6*A*), containing two phosphoserine residues incorporated chemically, is nearly identical to the degron in IκBα (Fig. 1*B*).

As schemed in Figure 6*B*, the chemoenzymatic ligation reaction is initiated by E1 that reacts with Ub to form an E1–S∼Ub thioester. Because E1 does not possess the basic residues necessary for deprotonating amine-based nucleophiles, the E1–S∼Ub intermediate readily reacts with nucleophilic thiols, such as GSH, to form an Ub α-thioester. Such Ub α-thioester then reacts with the synthetic Cys-β-catenin peptide, resulting in formation of Ub–Cys-β-catenin. Because Ub carries fluorophore iFluor 647 (Fig. 1*C*), Ub (I647)–β-catenin can be tracked by fluorescence imaging.

**Figure 7:**
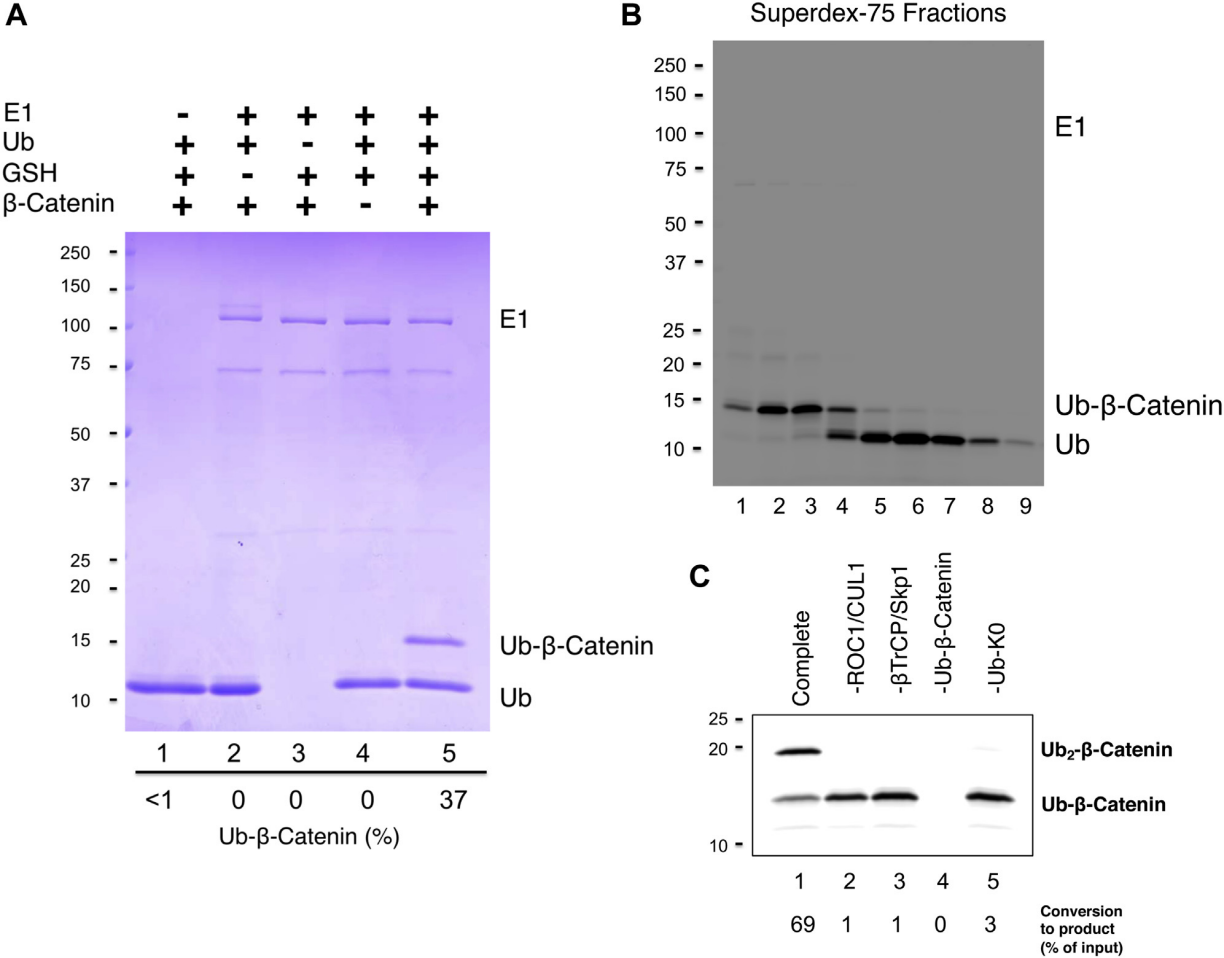
**Preparation of Ub–β-catenin active in ubiquitination.***A*, ligation reaction. The reaction was carried out as described in Experimental procedures section. Reaction products were imaged by Coomassie stain. The percentage of Ub converted to Ub–β-catenin is indicated. *B*, purification of β-catenin–Ub. Indicated fractions from chromatography through FPLC superdex-75 were separated by SDS-PAGE followed by imaging iFluor 647 using the Typhoon scanner. Ub–β-catenin and unincorporated Ub were well separated. *C*, Ub–β-catenin supports ubiquitination. The reaction was carried out as described in Experimental procedures section. Only in complete reaction (lane 1), Ub–β-catenin was conjugated with Ub–K0 to form Ub_2_–β-catenin. Ub, ubiquitin.

Incubation of the Cys-β-catenin degron peptide with Ub in the presence of E1 and GSH formed Ub–β-catenin with 37% efficiency (Fig. 7*A*, lane 5). Removal of E1 or GSH abolished the reaction (Fig. 7*A*, lanes 1 and 2). As shown, Ub–β-catenin was readily separated from unincorporated Ub and E1 by gel filtration (Fig. 7*B*). When the purified Ub–β-catenin was subjected to ubiquitination assay, nearly 70% of this fusion substrate was consumed, forming the Ub_2_–β-catenin product (Fig. 7*C*, lane 1). This reaction required intact SCF^βTrCP^ (Fig. 7*C*, lanes 2 and 3).

**Figure 8:**
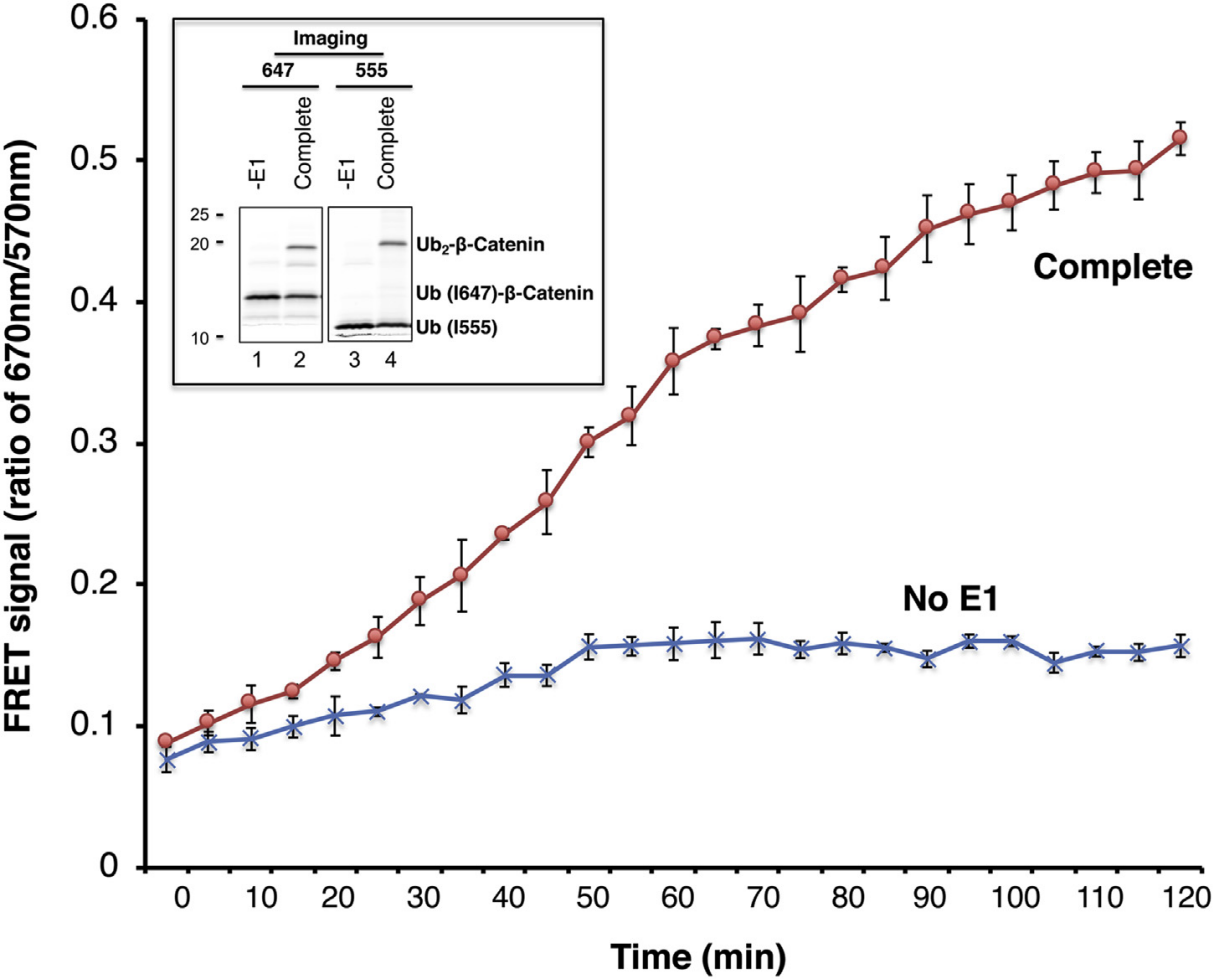
**FRET analysis of Ub–β-catenin.** The real-time kinetic reaction was carried out as described in Experimental procedures section. The inset shows gel images of the final reaction products detected by scanning with Typhoon FLA9500 for both iFluor 647 and iFluor 555, respectively. Approximately 28% of input Ub (I647)–β-catenin was converted into Ub_2_–β-catenin. Ub, ubiquitin.

To assess FRET β-catenin diubiquitination in real time, SCF^βTrCP^/Ub (I647)–β-catenin and Cdc34∼Ub (K48R/I555), preassembled in separate tubes, were mixed in a 384-well plate followed by incubation at 30 °C on the fluorescence reader. Real-time fluorescence was recoded at every 5 min interval as the ubiquitination proceeded. The results revealed that FRET increased over 120 min (Fig. 8). As shown by gel analysis, ∼30% of the input donor Ub was converted into the product after 120 min of incubation (Fig. 8, inset).

## Discussion

### Applicability of the FRET diubiquitination system

This work has established the FRET-based diubiquitination system. This platform produces a uniform di-Ub product depending on specific substrate–E3 interactions, such as those between E3 SCF^βTrCP^ and IκBα or β-catenin phosphodegron (Figs. 1 and 6). The diubiquitination creates proximity between the Ub-linked donor and acceptor fluorophores, respectively, resulting in energy transfer to yield a distinct FRET signal (Figs. 2 and 8). In principle, a defined E3-binding segment from any substrate can be tested in the FRET diubiquitination system as long as its cognate E3 ligase, in purified form, can be obtained and can cooperate with a K48-specifc E2 enzyme such as Cdc34. The FRET diubiquitination relies on Ub–substrate fusion, which can be implemented using either one of the two validated strategies. First is the use of recombinant Ub fusion. This method can apply to all substrate peptides that in their unmodified forms can bind to E3, or with modifications that can be mimicked by substituted amino acids, such as IκBα (EE)–Ub (Fig. 1*B*). The second strategy is chemoenzymatic ligation, which we have used to generate Ub–β-catenin (Fig. 6). While the recombinant Ub fusion method is cost effective with high yield, the chemoenzymatic ligation approach may be applicable for any substrate degron requiring a form of chemical modification that cannot be readily mimicked by amino acid substitutions.

There are limitations associated with the FRET diubiquitination system. First, the chemoenzymatic ligation method requires a degron of small size, preferably in the range of ∼20 amino residues, such as the β-catenin degron peptide (Fig. 6*A*). This is because the E3–E2 complex anchored on the degron motif, such as the IκBα/β-catenin DpSGXXpS box, must be positioned near the K48 residue from the receptor Ub that is fused to the substrate degron. Such structural constrain limits the optimal spacing between the degron motif and the attacking lysine to be approximately 13 to 14 amino acid residues (21). Second, because the FRET fluorophore pair is chosen based on the K48 di-Ub structural model (Fig. 1*C*), the current diubiquitination assay most likely works with K48-specific E2s only, such as Cdc34. However, modifications can be made to accommodate diubiquitination of non-K48 linkage based on available structural models of Ub chains of various linkages (27).

### FRET diubiquitination measures ubiquitination in real time

The FRET diubiquitination platform measurers ubiquitination in real time under both multiple-round (Fig. 3) or single-round turnover (Fig. 4) conditions. Moreover, the reaction was susceptible to competitive inhibition (Fig. 5). These features may prove advantageous in studying ubiquitination dynamics concerning dynamic assembly and disassembly of complexes containing enzymes (E2/E3), Ub, and substrate. Currently, dynamics studies are limited because traditional methods typically monitor ubiquitination in test tubes by visualizing products resolved on denaturing polyacrylamide gels. In contrast, the FRET diubiquitination is robust and proceeds in a real-time format. Moreover, a fluorescence plate reader can be equipped with an automated dispenser that can dispense reagents to reactions at any desired time through injection with minimal disturbance. These features should allow the analysis of ubiquitination at levels of sensitivity and precision unmatched by conventional gel analysis.

### FRET diubiquitination: a basis for HTS

The FRET diubiquitination platform uses a pair of matched fluorophores that are linked to the donor and receptor Ub, respectively, as illustrated in Figure 1*C*. Using this pair of probes, we have recently carried out an HTS and identified suramin that blocks the recruitment of E2 Cdc34 to the E3 ROC1–CUL1 subcomplex (20). Thus, it should be feasible to use the FRET diubiquitination reporter system as a platform for a powerful and activity-based HTS campaign to discover small-molecule inhibitors or activators that target substrate–E3 interaction. One attractive application of the FRET diubiquitination-based HTS would be to identify small-molecule antagonists that could block the ubiquitination of tumor suppressors and agonists that could stimulate the ubiquitination of oncoproteins. There is a growing list of tumor suppressors and oncogene products targeted by the Ub–proteasome system (28). As reviewed by Skaar *et al.* (5, 6), E3 SCF targets an array of oncogene products, such as β-catenin (by SCF^βTrCP^) and c-Myc (by SCF^Fbxw7^), as well as tumor suppressors, such as p27 and p21 (by SCF^Skp2^). Studies with auxin-regulated SCF^TIR1^–Aux interactions (29) and lenalidomide mediated CRL4^CRBN^ E3 Ub ligase activity (8, 9, 10) have provided proof-of-principle evidence for enhanced ubiquitination by small molecule probes.

## Experimental procedures

### Ub preparation

Yeast Ub in pET3a with an N-terminal tobacco etch virus cleavable His_6_-tag (pHisTEVyUb) was used as the template plasmid. His–Ub–Q31C/K48R was constructed, expressed, purified, and labeled with iFluor 555 maleimide (AAT Bioquest) as described previously (20). Purified, labeled His–Ub–Q31C/K48R–I555 was used as a donor Ub throughout this study, unless indicated otherwise. His–Ub–E64C was similarly constructed, expressed, and purified. His–Ub–E64C was used for ligation with the β-catenin peptide degron in experiments shown in Figure 6, Figure 7, Figure 8.

### Preparation of IκBα (EE)–Ub (E64C)–I647

The construction, expression, and purification of glutathione-*S*-transferase (GST)–IκBα (EE)–Ub (E64C) was described previously (20) (used in Figure 2, Figure 3, Figure 4, Figure 5). To prepare fluorescent IκBα (EE)–Ub (E64C)–I647, purified GST–IκBα (EE)–Ub (E64C) (20 mg) was treated with 10 mM tris(2-carboxyethyl)phosphine (TCEP) (Sigma) in degassed PBS for 15 min at room temperature, and then bound to Glutathione Sepharose beads (GE; 0.8 ml) for 1 h at 4 °C. After binding, the beads were washed with degassed PBS to remove TCEP and any unbound protein. For labeling, the beads were incubated with iFluor 647 maleimide (100 μl; AAT Bioquest; dissolved in *N*,*N* dimethylformamide [Sigma]) and PBS in a final volume of 800 μl, with rotation, for 1 h at room temperature and then overnight at 4 °C, in the dark. Note that the aforementioned handling was performed inside an anerobic glove box (Captair Pyramid, Erlab) under nitrogen gas. The subsequent steps were performed in normal air. Following incubation, the beads were washed to remove unreacted dye, and then subjected to digestion by biotin–thrombin (10 U), with rotation, for 4 h at room temperature and then overnight at 4 °C. Streptavadin beads (215 μl) were then added to capture biotin–thrombin by rotation for 1 h at 4 °C. Supernatant was saved. The remaining beads were washed with buffer A (25 mM Tris–HCl, pH 7.5, 10% glycerol, 0.01% NP-40, and 1 mM DTT) plus 50 mM NaCl four times, 0.4 ml/time. The first two washes were saved and pooled with the supernatant, resulting in GST-free IκBα (EE)–Ub (E64C)–I647. This material was filtered to remove residual beads and dialyzed against 1 L of buffer A plus 50 mM NaCl. The final IκBα (EE)–Ub (E64C)–I647 preparation was 0.7 mg/ml with a yield of 1.12 mg.

### Ligation of the β-catenin degron peptide to Ub by chemoenzymatic reaction

Reaction mixture (10 μl) contained 50 mM Tris–HCl, pH 7.5, 5 mM MgCl_2_, 2 mM ATP, 20 mM GSH (pH adjusted to 7.0), 0.5 μM His–E1, 12.5 μM His–Ub (E64C), and 75 μM β-catenin degron peptide (NH2-CAWQQQSYLD(pS)GIH(pS)GATTTAP-OH; purchased from New England Peptide Inc) (Fig. 7*A*). The reaction was at 37 °C for 2 h. The reaction mixture was separated by 4 to 20% SDS-PAGE and visualized by Coomassie stain.

### Preparation of Ub (E64C)–I647–β-catenin

Ligation of the β-catenin degron peptide to Ub (E64C), as described previously, was scaled up to 4.2 ml (used in Figs. 7*C* and 8). The entire reaction mixture was treated with 10 mM TCEP and then bound to Ni–nitrilotriacetic acid agarose (Qiagen; 0.1 ml) for 1 h at 4 °C. The beads captured His–Ub (E64C)–β-catenin, unconjugated His–Ub (E64C), and His–E1. After binding, the beads were washed with degassed PBS to remove TCEP and any unbound protein. The resulting beads were then incubated with iFluor 647 maleimide (10 μl in 500 μl of PBS), with rotation, for 1 h at room temperature and then overnight at 4 °C, in the dark. Note that the aforementioned handling was performed inside an anerobic glove box. The subsequent steps were performed in normal air. Following incubation, the beads were washed with PBS and then eluted with 250 mM imidazole in PBS (500 μl). The eluted material was filtered and dialyzed against 1 L of buffer A plus 50 mM NaCl.

To remove His–E1 and His–Ub (E64C)–I647, the aforementioned material was chromatographed through Superdex 75 (FPLC). The column was eluted with 500 ml of buffer A plus 150 mM NaCl. Fractions were collected and analyzed through gel electrophoresis followed by imaging iFluor 647 using the Typhoon FLA 9500 laser scanner (GE). As shown in Figure 7*B*, Ub (E64C)–I647–β-catenin and Ub (E64C)–I647 were well separated. Fractions containing Ub (E64C)–I647–β-catenin were pooled and used for ubiquitination experiments shown in Figures 7*C* and 8. The yield of Ub (E64C)–I647–β-catenin was 833 pmol (∼10 μg) at 4.2 μM out of 56 nmol of His–Ub–E64C used.

### Other reagents

The following reagents were prepared using established protocols. Skp1–βTrCP, ROC1–CUL1, PK–Ub, HA–Cdc34, and Ubc12 were prepared using methods described by Chong *et al*. (30). Nedd8–ROC1–CUL1 is a gift from N. Zheng of University of Washington, Seattle. Nedd8, Nedd8 E1, and human Ub–K0 were purchased from Boston Biochem.

### Analysis of the FRET IκBα diubiquitination in test tube

#### Neddylation of ROC1–CUL1

The mixture contained 50 mM Tris–HCl, pH 7.4, 5 mM MgCl_2_, 2 mM ATP, 0.5 mM DTT, 0.1 mg/ml bovine serum albumin, ROC1–CUL1 (0.45 μM), Nedd8 (20 μM), Nedd8 E1 (83 nM), and Ubc12 (15 μM) (Fig. 2). The reaction was incubated at room temperature for 10 min.

#### Assembly of the SCF^βTrCP^/IκBα (EE)–Ub (E64C)–I647 complex

Skp1–βTrCP (0.45 μM) was added to the aforementioned mix. Incubation continued at room temperature for 10 min. IκBα (EE)–Ub (E64C)–I647 (5 μM) was added, and the reaction volume was adjusted to 5 μl. The reaction was incubated for additional 10 min at room temperature.

#### Assembly of Cdc34∼Ub (K48R/Q31C)–I555

The E2 charging reaction was assembled in a mixture (5 μl) that contained 50 mM Tris–HCl, pH 7.4, 5 mM MgCl_2_, 2 mM ATP, 0.5 mM DTT, 0.1 mg/ml bovine serum albumin, Ub (K48R/Q31C)–I555 (6.6 μM), E1 (0.1 μM), and human Cdc34 (10 μM). The reaction was incubated for 5 min at 37 °C.

#### IκBα diubiquitination

The aforementioned mixture containing SCF^βTrCP^/IκBα (EE)–Ub (E64C)–I647 or Cdc34∼Ub (K48R/Q31C)–I555 was mixed (a final volume of 10 μl). The reaction was incubated at 37 °C for times as indicated. For gel analysis, an aliquot of the products was separated by 4 to 20% SDS-PAGE and visualized by imaging using the Typhoon FLA 9500 laser scanner (GE). For spectroscopic analysis, an aliquot of the reaction mixture (1 μl) was mixed with 30 μl of PBS, and the mixture was spotted onto a 384-well plate, which was read by the Synergy-H1 reader (BioTek).

### Real-time analysis of FRET IκBα diubiquitination

For experiments shown in Figures 3, *A*–*C*, 4, and 5, *A*–*B*, SCF^βTrCP^/IκBα (EE)–Ub (E64C)–I647 complex and of Cdc34∼Ub (K48R/Q31C)–I555 were each assembled in test tube as described in section “*Analysis of FRET IκBα diubiquitination in test tube*,” except that the reaction volume of 10 μl was used. These two mixes were combined in a well on a 384-well plate (Figure 3, Figure 4, Figure 5). The incubation proceeded at 30 °C on the Synergy-H1 reader, and the reaction was monitored at times as indicated. The results are presented by either an emission spectrum of 544 to 700 nm (Figs. 3*A*, 4, and 5*A*) or a ratio of 670/570 nm (Figs. 3*C* and 5C) that represents the FRET efficiency.

For experiments shown in Figures 3, *D* and *E* and 5*C*, Nedd8–ROC1–CUL1 was used, and hence, the neddylation reaction was omitted. The concentrations of enzymes and Ub are indicated in the legends to these figures. The results are presented by the ratio of 670/570 nm.

### Analysis of FRET β-catenin diubiquitination

For the reaction shown in Figure 7*C*, it was carried out essentially the same way as described in “*Analysis of FRET IκBα diubiquitination in test tube*.” The exceptions are that Ub–I647–β-catenin (0.3 μM), neddylated SCF^βTrCP^ (0.9 μM), Ub–K0 (20 μM), and Cdc34 (3 μM) were used. The reaction was at 37 °C for 30 min. The reaction mixture was separated by SDS-PAGE and visualized by imaging with the Typhoon scanner.

For the reaction shown in Figure 8, it was carried out essentially the same way as described in “*Real time analysis of FRET IκBα diubiquitination*.” The exceptions are that Ub–I647–β-catenin (0.3 μM), neddylated SCF^βTrCP^ (0.9 μM), donor Ub (C31, K48R, and I555; 1 μM), and Cdc34 (3 μM) were used. The reaction, assembled on a 384-well plate, proceeded at 30 °C on the Synergy-H1 reader and was monitored at times as indicated. The ratio of 670/570 nm, representing the FRET efficiency, is presented.

## Data availability

All data are contained within the article.

## Conflict of interest

The authors declare that they have no conflicts of interest with the contents of this article.
